# Exploring the Effects of Short-Term Daily Intake of *Nitraria retusa* Tea on Lipid Profile: A Pre-Post, Uncontrolled Pilot Study in Both Healthy and Overweight/Obese Adults

**DOI:** 10.3390/nu15163649

**Published:** 2023-08-20

**Authors:** Aicha Laouani, Hana Nasrallah, Awatef Sassi, Farhana Ferdousi, Feten Zar Kalai, Yosra Hasni, Khalifa Limem, Hiroko Isoda, Saad Saguem

**Affiliations:** 1 Laboratory of Metabolic Biophysics and Applied Pharmacology, Faculty of Medicine, University of Sousse, Sousse 4002, Tunisia or aicha.laouani@famso.u-sousse.tn (A.L.);; 2 USCR Analytical Platform UHPLC-MS & Research in Medicine and Biology, Faculty of Medicine, University of Sousse, Sousse 4002, Tunisia; 3 Faculty of Life and Environmental Sciences, University of Tsukuba, Tsukuba 305-8572, Japan; 4 Alliance for Research on the Mediterranean and North Africa (ARENA), University of Tsukuba, Tsukuba 305-8572, Japan; 5 Laboratory of Aromatic and Medicinal Plants, Center of Biotechnology, Technopark of Borj Cedria, BP 901, Hammam-Lif 2050, Tunisia; 6 Endocrinology-Diabetology Department, Farhat Hached Hospital, Sousse 4003, Tunisia; 7 Department of Biochemistry, Faulty of Medicine, University of Sousse, Sousse 4002, Tunisia; 8 Open Innovation Laboratory for Food and Medicinal Resource Engineering (FoodMed-OIL), National Institute of Advanced Industrial Science and Technology (AIST), Tsukuba 305-8577, Japan

**Keywords:** *Nitraria retusa*, HDL, triglyceride, overweight, obesity, dyslipidemia

## Abstract

In the present study, we aimed to explore the feasibility, compliance, and potential benefits of *Nitraria retusa* extract (NRE) intervention in both healthy (BMI ≤ 24.9 Kg/m^2^) and overweight/obese adults (BMI > 25 Kg/m^2^). A total of 98 participants, including 37 healthy individuals and 61 overweight/obese adults, were randomly assigned to either a low-dose (500 mg/day) or a high-dose (2000 mg/day) NRE intervention group. Plasma lipid biomarkers, liver and kidney functions, general hematology, and blood glucose levels were measured at the baseline and 10 days after intervention. While the lipid profile of the healthy participants did not show any statistically significant changes, the obese participants in the high-dose group experienced a significant decrease in triglyceride levels (within-group difference *p* value = 0.004) and an increase in HDL levels (within-group *p* value < 0.001). No significant differences were observed in other parameters, indicating that NRE at the given doses was safe. Furthermore, the study had impressive compliance and acceptability, with over 90% of participants completing the intervention and diligently following the study protocol. This pilot study represents the first investigation into the feasibility, acceptability, and potential benefits of NRE intervention on lipid profiles in human volunteers.

## 1. Introduction

Dyslipidemia is characterized by a decrease in plasma high-density lipoprotein cholesterol (HDL) or an increase in one or a combination of plasma triglyceride (TG), total cholesterol (TC), and low-density lipoprotein cholesterol (LDL) levels [1]. The prevalence of hypercholesterolemia varies across regions, ranging from 22.6% to 54% in Africa, Asia, Europe, and America, according to the World Health Organization [2]. In Tunisia, the prevalence of dyslipidemia in the population is 44% [3]. Dyslipidemia is implicated in various diseases, including cardiovascular disease, diabetes, and obesity [4]. Literature reports indicate that 1.3–47.8% of adult North Africans are overweight or obese [5]. In Tunisia, according to a report in 2010, 62% of adults are overweight, and 28% of the adult population is obese [6]. Elevated body mass index (BMI) consistently correlates with increased TG, TC, and LDL levels, as well as decreased HDL levels [7]. The most significant contributing factor for obesity-related dyslipidemia is the increased free fatty acid release from adipose tissue to the liver. The enhanced free fatty acid release was proposed to lead to increased TG [8] and very-low-density lipoprotein (VLDL) production and can inhibit lipolysis, thereby promoting hypertriglyceridemia [9]. Moreover, lipolysis of triglycerides and LDL receptor expression is impaired in obesity. This will ultimately affect HDL metabolism, leading to lower levels of HDL-C with impairment of reversed cholesterol transport [8]. This correlation between BMI and dyslipidemia has been proposed as a risk factor for cardiovascular diseases among obese individuals [10].

While medications are commonly used to reduce the risk of dyslipidemia, they often have side effects such as liver injury, flushing, headaches, and elevated LDL levels [11]. Therefore, the search for safer alternatives for the prevention and treatment of dyslipidemia is warranted. Plants offer a promising avenue due to their accessibility, safety, and effectiveness, potentially serving as sources for new drugs with minimal adverse effects [12]. Several medicinal plants used in traditional medicine have been suggested as potential means to prevent dyslipidemia without significant side effects [13,14]. For instance, the consumption of green tea for 12 weeks has been shown to decrease TC and LDL levels in women with abdominal obesity without any adverse effects [15]. Additionally, the intake of *Emblica officinalis* twice daily for 12 weeks significantly reduced TG, TC, and LDL levels in participants with dyslipidemia [13].

In this context, *Nitraria retusa*, a salt-tolerant plant belonging to the *Nitrariaceae* family [16], is primarily found in Asia, North Africa, Russia, and Europe [17]. In Tunisia, it grows in the southern regions and is locally known as Ghardaq [18]. The leaves of *Nitraria retusa* have been traditionally used in folk medicine for treating hypertension and inflammation [16,19]. Additionally, *Nitraria retusa* has been reported to possess antioxidant, anticancer, antimicrobial, and antiviral activities [20,21,22]. Studies have revealed that the leaf extract of *Nitraria retusa* contains significant quantities of tannins, alkaloids, steroids, and flavonoids, which may be responsible for its beneficial effects [23,24]. Furthermore, an investigation conducted on 3T3-L1 cells revealed that *Nitraria retusa* extract (NRE) inhibits differentiation, reduces cell size and number, and decreases fat accumulation and lipid droplet content [23]. Previous research has demonstrated that *Nitraria retusa* regulates the expression of genes involved in lipolysis and lipogenesis, promoting lipid metabolism in the liver of obese mice [23,24]. 

However, to the best of our knowledge, no clinical studies have been conducted using NRE. Therefore, in this pilot study, we aimed to assess the feasibility, acceptability, and preliminary effects of NRE intervention on lipid profiles in both healthy and overweight/obese adults. 

## 2. Materials and Methods

### 2.1. Plant Material 

*Nitraria retusa* was collected from saline soils in Sabkha El Kelbia, a region situated 14 km to Kairouan, in mid-Tunisia, on 2 March 2021. The leaves (aerial parts) were rinsed with water, dried in a dark, ventilated room at ambient temperature for 15 days, and finely ground using a mill-type Moulinex (MOULINEX SA, Paris, France). The plant material obtained was stored in tightly closed containers for further use.

### 2.2. Preparation of NRE and Quantification of Its Active Compounds 

To prepare the NRE infusion, the dried leaf powder was steeped in 100 mL of boiling water for 15 min. The NRE dose was standardized using a validated high-performance liquid chromatography (HPLC) method (UHPLC-DAD, SHIMADZU 8045, Kyoto, Japan), encompassing qualitative and quantitative analysis. Two doses were identified and administered: a low dose containing 5 mg of flavonoids with 4 µg of isorhamnetin and a high dose containing 20 mg of flavonoids with 16 µg of isorhamnetin.

### 2.3. Study Settings and Recruitments

This study was conducted at the Laboratory of Biophysics, Faculty of Medicine, University of Sousse, and the Department of Endocrinology and Diabetology, Farhat-Hached University Hospital, Sousse, Tunisia. The participants were recruited across the Sousse governorate area between March 2021 and April 2022. Participants were recruited from different Tunisian regions through the personal contacts of interviewers and their relatives and by using direct approaches in public spaces. 

### 2.4. Study Participants 

A total of 111 volunteers were initially screened; among them, 98 individuals met the eligibility criteria for the study—37 healthy subjects and 61 overweight/obese participants. The healthy subjects were randomly assigned to either the low-dose group (n = 18) or the high-dose group (n = 19). Similarly, the overweight and obese participants were also assigned to either the low-dose group (n = 23) or the high-dose group (n = 38). Study participants were asked to keep their same daily routine during the trial period.

#### 2.4.1. Inclusion Criteria 

Both male and female participants between the ages of 18 and 75 were recruited for this study. The BMI criteria for inclusion were ≤24.9 kg/m^2^ for healthy individuals and >25 kg/m^2^ for overweight and obese participants and participants who provided informed consent by signing a consent form.

Although our primary consideration was an equitable representation of both sexes to ensure a balanced demographic, we had more women volunteers for the study than male volunteers due to women’s higher level of interest in the antiobesity trial. 

#### 2.4.2. Exclusion Criteria

The following criteria were used to exclude participants from the study: professional athletes, smokers, pregnant or breastfeeding individuals, those with any medical or psychiatric disorders or chronic conditions, a history of weight-reducing surgery or eating disorders, and individuals with food allergies. Participants with a history of cardiovascular disease, hypertension, diabetes, asthma, or major gastrointestinal problems and taking medication known to affect lipid metabolism were also excluded. 

### 2.5. Study Design, Assessments, and Study Outcomes

This study used a prospective pre-test/post-test design. Both healthy (BMI ≤ 24.9 kg/m^2^) and overweight/obese (>25 kg/m^2^) groups of participants were randomly assigned to either high- or low-dose groups.

Over a span of ten consecutive days, study participants consumed *Nitraria retusa* tea prepared by steeping either 2000 mg or 500 mg of finely powdered leaves in 100 mL of boiled water for a duration of 15 min, after which it was filtered through a strainer. This process was performed once every day. Volunteers enrolled in the study were informed of the importance of keeping their same daily routine during the trial period. All participants were aware of the necessity of following the instructions they received before the study.

During the initial screening, participants underwent interviews that included a comprehensive assessment of their medical and nutritional history, as well as measurements of weight, height, and BMI. Vital signs such as blood pressure and pulse rate were also assessed.

Fasting blood samples were collected using three different types of tubes: heparin-coated, EDTA-coated, and sodium fluoride/potassium oxalate-coated tubes. These samples were then centrifuged (20 min, 3000 rpm, 4 °C) and stored at −80 °C until further analysis. All parameters were assessed using the UniCel DXC^®^ 600 Synchron by Beckman Coulter™ analyzer at the Biochemistry Department of Farhat Hached Hospital, Sousse, Tunisia. Measurements and blood sample collection were taken at two time points: baseline and after 10 days of intervention. All measurements were conducted after an overnight fast (8–9 h) using standardized methods.

Liver and kidney function tests involved the assessment of parameters alkaline phosphatase (ALP), serum albumin (sAlb), alanine aminotransferase (ALT), gamma-glutamyltransferase (GGT), total bilirubin (BT) and direct bilirubin (BD), aspartate aminotransferase (AST), blood urea nitrogen (BUN), and serum creatinine (sCr). 

Lipid profiles, including TG, TC, and HDL, were measured using the Beckman Coulter™ analyzer (UniCel DXC^®^ 600) following the principle of spectrophotometric techniques. Blood sugar was also measured potentiometrically using the Beckman Coulter D600 analyzer. LDL cholesterol was calculated using the Friedewald formula (LDL= [TC − HDL] − TG/5). 

General hematology parameters were also measured, such as white blood cell (WBC) count, red blood cell (RBC) count, hemoglobin (Hb), hematocrit (Ht), and platelet (Plt) count.

### 2.6. Statistical Analysis

Data are presented as mean ± standard deviation (SD). Normal distributions of the data were assessed by the Shapiro–Wilk test. The homogeneity of variance was measured using Levene’s test. Within-group differences in the parameters before and after the intervention were compared using a paired *t*-test (for normally distributed data) and Wilcoxon signed rank test (nonparametric data). Between-group differences (low dose vs. high dose) in the parameters were assessed by using an independent samples *t*-test (parametric and equal variances), Welch’s *t*-test (unequal variances), and Mann–Whitney U test (nonparametric). Results were considered significant at *p* < 0.05. All statistical analyses were performed using SPSS 28.0 for Windows (IBM Corp., New York, NY, USA).

### 2.7. Ethical Consideration

The study protocol was approved by the Human Research Ethics Committee at the Faculty of Medicine, University of Sousse, Tunisia, with Ethical Approval Number CEFMS 34/2019. Informed consent was obtained from all participants involved in the study. 

## 3. Results

### 3.1. Enrollment of Study Participants

**Figure 1:**
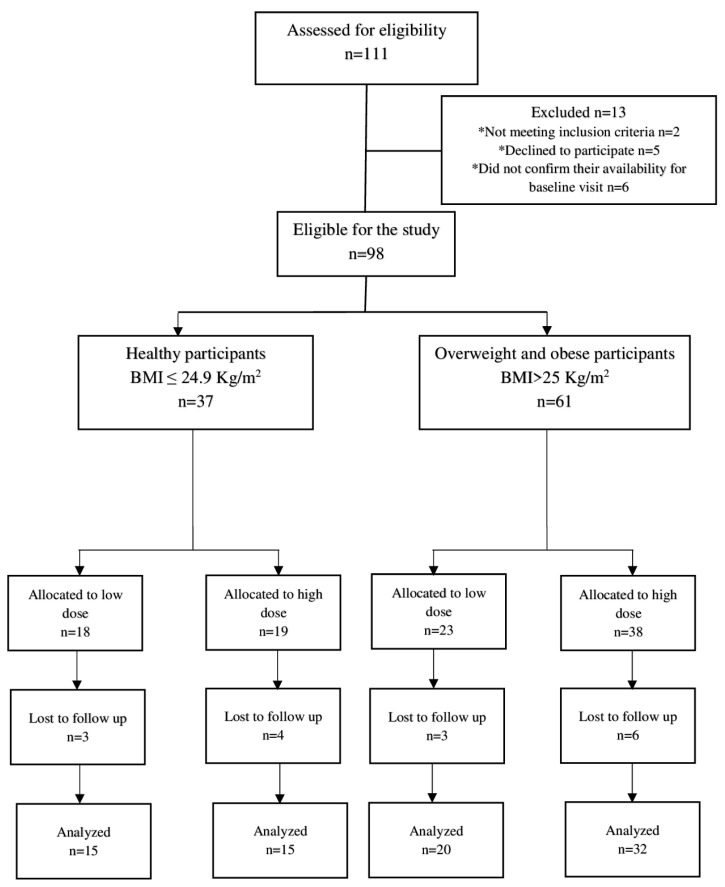
Trial flow chart of the study. * Reasons for exclusion from the study.

A total of 111 participants were initially screened for the study. Among them, 13 individuals were deemed ineligible for enrollment, with two not meeting the inclusion criteria, five declining to participate, and four failing to confirm their availability for the baseline visit. Ultimately, 98 participants, including 37 healthy and 61 overweight/obese individuals, were enrolled and allocated into low- and high-dose groups after providing informed consent. Among these participants, 82 individuals, comprising 30 healthy and 52 overweight/obese individuals, completed the 10-day intervention and were included in the efficacy analysis. The study demonstrated high compliance, with over 90% completing the intervention, thus highlighting their high acceptability of the NRE intervention. The trial flow chart of the study can be found in Figure 1.

### 3.2. Baseline Characteristics 

The baseline characteristics of the healthy and overweight/obese participants are presented in Table 1; Table 2, respectively.

A significant difference (*p* = 0.013) in age was observed among the healthy participants, with the low-dose group having a higher average age than the high-dose group. However, there were no significant differences in other baseline measurements, including sex, height, weight, and BMI, among the healthy participants (Table 1). Similarly, no significant differences in any baseline parameters were observed between the low- and high-dose groups in the overweight/obese participants (Table 2).

### 3.3. Safety Assessment of NRE Infusion 

Both doses of NRE infusions were found to be well tolerated. Mild undesirable effects were observed in three overweight/obese participants from the high-dose group, including temporary alterations in bowel movements, a diuretic impact lasting for two days, and periodic acid reflux. 

To assess safety, we examined the effect of daily consumption of NRE on various indicators such as hematological and hemodynamic parameters, liver and kidney function tests, and blood glucose levels. All participants exhibited normal blood biochemical values during the screening phase for study enrollment. The results are presented in Table 3; Table 4.

Based on our findings, there were no significant differences observed in RBC count, WBC count, Hb level, Ht, and Plt count between the groups receiving two different doses of NRE for 10 days. Furthermore, the hemodynamic parameters, including systolic blood pressure (SBP), diastolic blood pressure (DBP), and pulse rate, exhibited no significant differences in healthy participants. However, overweight/obese participants showed a lower SBP in both groups and a lower DBP in the high-dose group after 10 days of intake of NRE, which remained within normal limits (<140 mmHg for SBP and <90 mmHg for DBP).

**Figure 2:**
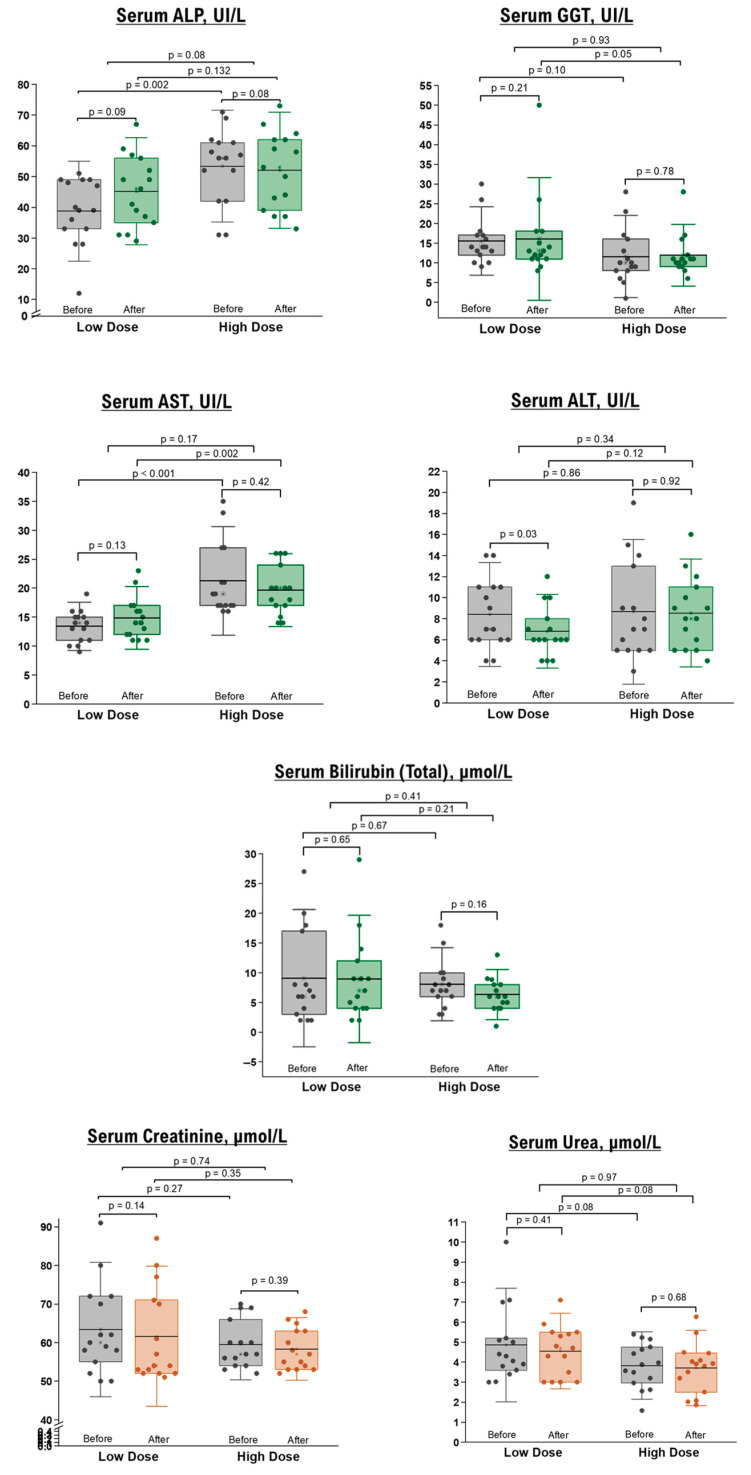
Liver and renal function tests in healthy participants before and after 10 days of NRE intervention. The box represents the percentiles, the error bar represents the SD, the midline represents the mean value, and the star symbol represents the median values.

Concerning hepatobiliary indices, including serum AST, BT, BD, sAlb, ALT, ALP, and GGT, no differences were observed post-intervention compared to the baseline measurements. However, there was a significant reduction in ALT levels in the low-dose group of healthy participants, which remained within the normal range (Figure 2).

Renal function tests, specifically BUN and sCr, demonstrated no significant differences before and after the intervention in healthy (Figure 2) and overweight/obese (Table 4) participants. Additionally, fasting blood sugar levels remained unchanged in both groups following the NRE intervention.

Our preliminary results suggest the safety of daily consumption of NRE in terms of liver and kidney functions, encompassing both healthy individuals and those who are overweight or obese.

### 3.4. Effects of NRE Intervention on Lipid Profiles 

**Figure 3:**
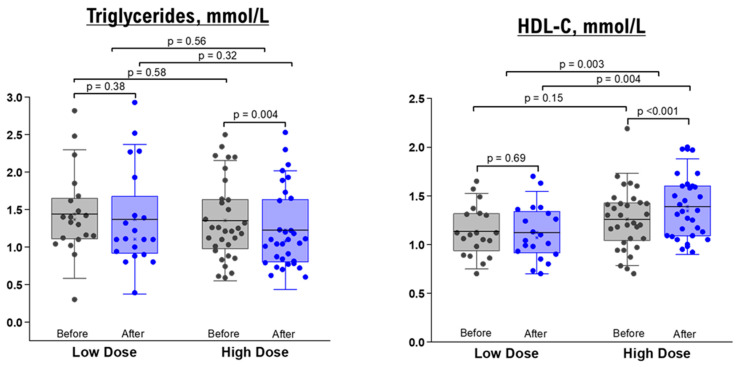
TG and HDL levels before and after 10 days of NRE intervention in overweight and obese participants. The box represents the percentiles, error bar represents the SD, midline represents the mean value, and the star symbol represents the median value.

As shown in Table 3; Table 4, the comparison between baseline measurements and those taken after a 10-day administration of NRE infusion revealed no significant changes in TG, TC, HDL, and LDL levels for both healthy and overweight/obese participants who received the low-dose intervention. Likewise, when it came to the high-dose groups, there were no significant differences in lipid profiles before and after the intervention among healthy participants (Table 3). Nevertheless, overweight/obese participants in the high-dose group displayed a significant decrease in TG level (within-group difference *p* value = 0.004) and an elevation in HDL level (within-group *p* value < 0.001, between-group comparison after intervention *p* value = 0.004, and between-group mean difference *p* value = 0.0003) (Table 4 and Figure 3).

We further conducted stratified analyses to examine how age and BMI could influence the response to the NRE intervention among overweight/obese participants in the high-dose group (as detailed in Supplementary Tables S1–S8). Our findings indicate that among participants with a BMI below 29.9, there was no noteworthy alteration observed in TG levels, while HDL demonstrated a marginally significant increase (*p* = 0.051) (Supplementary Table S1). However, among participants with a BMI exceeding 30.0, both TG and HDL parameters exhibited significant changes post-NRE intervention—TG levels showed a significant decrease (*p* = 0.006), and HDL levels displayed a significant increase (*p* < 0.001) (Supplementary Table S2). Moreover, among the overweight/obese participants in the high-dose group, there was a consistent and significant elevation in HDL levels, observed both in individuals aged below 40 years (*p* = 0.005) and those aged above 40 years (*p* = 0.001) following the NRE intervention. On the other hand, the decrease in TG levels was significant only within the younger age group (*p* = 0.01). Specifically, in participants of the high-dose group with high BMI (>30.0), the elevation in HDL levels remained significant in both the younger (<40 years) and older (>40 years) age categories (Supplementary Tables S6 and S8). Therefore, a follow-up trial specifically targeting overweight and obese individuals with significant dyslipidemia provide a more comprehensive evaluation of NRE’s actual potential.

## 4. Discussion

In the present study, we have reported the tolerability, acceptability, and safety of the short-term daily intake of NRE infusion in adults. Importantly, our results indicate that NRE might offer potential benefits for improving lipid profiles, particularly in overweight and obese individuals, highlighting the importance of conducting future controlled, randomized, large-scale clinical trials to validate and further explore these findings. Our study is the first to provide valuable insights into the potential antihyperlipidemic benefits of NRE in human volunteers.

The results obtained from this study suggest that the administration of NRE did not negatively affect the hepatic and renal balance. Moreover, there were no statistically significant deviations observed in any other hematological parameters, providing further evidence for the safe daily consumption of NRE infusion. Participants exhibited high compliance, with no reported problems or complications related to the daily ingestion of 2000 mg of NRE. However, a subset of overweight and obese participants did report experiencing transient laxative and diuretic effects for a limited duration. Furthermore, the antihypertensive effect of NRE is not well explored. In fact, few studies mentioned that the fruits and leaves of Nitraria have been used as a nutritional food and traditional herb for the treatment of hypertension and abnormal menstruation [19,24]. Further studies should be conducted to explore the antihypertensive effect of the infusion of crushed, dried leaves of *Nitraria retusa* in overweight/obese participants with elevated blood pressure levels (systolic blood pressure (SBP) ≥ 140 mmHg and/or diastolic blood pressure (DBP) ≥ 90 mmHg). Ambulatory blood pressure monitoring should be applied instead of standard one-time measurement.

Overall, the findings of this study establish the safety and tolerability of NRE in healthy adults following a 10-day consumption period. 

Additionally, we investigated the impact of the continuous intake of *Nitraria retusa* infusion on the lipid profile of overweight/obese participants. In an obese state, the enlarged adipose tissue induces an excessive release of free fatty acids leading to hypertriglyceridemia and reduced HDL levels [25]. Moreover, lipolysis of triglycerides and LDL receptor expression is impaired in obesity, which affects HDL metabolism, leading to lower levels of HDL-C with impairment of reversed cholesterol transport [8].

Our findings revealed that a higher dosage of NRE resulted in a decrease in TG levels and an increase in HDL levels (Figure 3). These outcomes align with a study conducted by Faten Zar Kalai et al., where they observed similar effects of NR ethanol extract in reducing TG levels and enhancing HDL cholesterol in an obese mice model over a 4-week period [23]. Another study provided further insights into the protective effects of *Nitraria retusa* ethanolic extract in obese mice by modulating the balance between lipolysis and lipogenesis [24]. Moreover, the age/BMI-stratified results proposed that the infusion of *Nitraria retusa* improved the lipid profile in younger, obese subjects compared to overweight elderly. These findings support other studies suggesting that HDL levels appeared to decrease with age, opposite to TG [26,27,28]. Thus, *Nitraria retusa* represents a promising source of new drug candidates for the prevention of dyslipidemia. 

The therapeutic effects of NRE can largely be attributed to the presence of various active compounds, including alkaloids, flavonoids, and coumarins. According to the previous study, the phenolic profile of *Nitraria Retusa* showed mainly high contents of isorhamnetin aglycone and glycosides [29] and other flavonoids like apigenin, quercetin, kaempferol, and luteolin [21]. These compounds may inhibit atherosclerosis [30] and were individually demonstrated to have a high potential to prevent metabolic syndrome diseases [31]. Nofer et al. showed that the reduction in triglyceride and total cholesterol LDL could be related to phenolic compounds [29]. The reduction in lipid biomarker levels may be due to the flavonoids and glycosides contained in *Nitraria retusa*. Flavonoids play an important role in the improvement of lipid profiles. Quercetin in an animal model caused a decrease in the level of LDL cholesterol and triglyceride [32]. Moreover, luteolin-7-O-glucoside and apigenin-7-O-glucoside have been found to reduce total cholesterol and triglyceride [33,34].

Active compounds such as alkaloids, flavonoids, and coumarins have demonstrated significant cardioprotective effects [35,36]. Additionally, the protective effects of *Nitraria retusa* can be attributed to its enhanced antioxidant status, facilitated by the phenolic components contained in the NR leaves, particularly isorhamnetin glycosides and aglycones [36].

Numerous in vitro and in vivo studies have reported the impact of isorhamnetin on obesity, highlighting its ability to reduce adipogenesis, restrict body fat accumulation, and improve glycemia and serum lipid profile [37,38]. Previous findings confirm that a study period of 10 days is methodologically valid. In fact, Chbili et al. proved that daily Laurus nobilis Tea Consumption for 10 days modulates lipid profile in healthy volunteers [39]. Khan et al. have shown that consuming bay leaves for 10 days could lead to varying serum lipid levels [40]. Moreover, Tazoho et al. showed that drinking calyx of sour tea for nine days reduced total cholesterol levels in healthy men [41]. Our results align with these studies, confirming the possible beneficial effect of the *Nitraria retusa* extract on lipid profile after 10 days of intake. 

In this study, the observed decrease in TG levels may be attributed to multiple mechanisms. Firstly, NRE may potentially inhibit the absorption of TG into the body while promoting its degradation and elimination through feces. Additionally, NRE may stimulate the transfer of TG to HDL. Numerous studies have highlighted the cardioprotective properties of HDL, including its ability to safeguard LDL from oxidative modification [42,43]. HDL is thought to exchange lipid peroxidation products between lipoproteins, as documented by Sanguinetti et al., who demonstrated that HDL prevents the formation of oxidatively modified LDL and protects against atherogenic diseases [42]. Another study proposed that HDL inhibits the accumulation of lipid peroxides on LDL, thus providing protection against atherosclerosis [44]. Moreover, HDL plays a crucial role in preventing the buildup of cholesterol, TGs, and LDL in the arteries by facilitating the transportation of cholesterol to the liver for elimination from the body. This process, known as reverse cholesterol transport, reduces the risks of conditions such as stroke and coronary artery diseases [45,46]. It is important to note that hypertriglyceridemia serves as an independent predictor of coronary artery disease [47], while high plasma TG levels are significant risk factors for type-2 diabetes [48] and insulin resistance [49]. 

At present, fibrates are widely prescribed as oral hypolipidemic agents in clinical practice for managing dyslipidemia [50]. While fibrates are generally well tolerated, their clinical use is often associated with certain adverse effects, such as an elevation in sCr level [51,52,53,54,55]. The findings of this study propose that *Nitraria retusa* could potentially serve as an alternative to synthetic hypolipidemic drugs, offering a promising option for lipid-lowering therapy.

In our study, we also evaluated the levels of TC and LDL as lipid biomarkers. No statistically significant changes in these lipid parameters were observed after daily consumption of NRE in both groups of participants, compared to their baseline levels. The inconsistent results may be attributed to this study group’s relatively lower baseline TC and LDL levels. Additionally, participants were instructed to maintain their regular diet without any restrictions, which suggests that the dietary components of their lifestyle may have had a more pronounced impact on plasma cholesterol levels than the NRE intervention, potentially introducing confounding effects. Furthermore, the lack of distinct differences in lipid biomarkers could also be attributed to the relatively short duration of the treatment period.

A more extensive and prolonged study, encompassing a larger number of participants from both genders within the same age range, is nevertheless warranted in order to achieve a more precise evaluation of the benefits conferred by NRE. Investigations of the benefic effects of *Nitraria retusa* tea on human lipidic status would also be strengthened by including a placebo group and a group with dyslipidemia. In parallel, conducting molecular docking and drug-likeness studies would provide valuable insights into the interactions between NRE and its potential target molecules. These studies can shed light on the pharmacological mechanisms underlying the hypolipidemic effects of NRE and help determine its suitability as a therapeutic agent.

## 5. Conclusions

In conclusion, our research revealed, for the first time, the safety and effectiveness of NRE infusion at a dosage of 2000 mg in reducing serum lipid levels in human volunteers within a brief 10-day period. Moreover, the infusion was well tolerated by overweight and obese participants, indicating its potential for future extended studies addressing dyslipidemia prevention and treatment in this group. These findings suggest that NRE may offer a safer alternative option compared to synthetic drugs, as it lacks significant adverse effects. To obtain comprehensive evidence and assess the efficacy of NRE infusion as a hypolipidemic agent, follow-up double-blind, randomized, placebo-controlled clinical trials specifically targeting overweight and obese individuals with significant dyslipidemia are needed, preferably with extended treatment duration.

## Figures and Tables

**Table 1:** Baseline characteristics of the healthy study participants.

		Low-Dose Group (n = 15)		High-Dose Group (n = 15)		*p* Value ^a^
Variables		Mean	(SD)	Mean	(SD)	
Age, y (range)		36.4	(25–56)	29.0	(19–59)	0.013
Sex, N (%)	Male	4	(26.7)	2	(13.3)	0.65
	Female	11	(73.3)	13	(86.7)	
Height, cm		164.8	(7.4)	168.4	(7.2)	0.58
Weight, kg		62.6	(8.8)	64.3	(8.1)	0.19
BMI, kg/m^2^		22.9	(1.8)	22.6	(1.7)	0.56

^a^ Between-group differences (low dose vs. high dose) in the parameters were assessed by using unpaired *t*-test (parametric) and Mann–Whitney U test (nonparametric) for continuous variables and Fisher’s Exact Test (2-sided) for categorical variables.

**Table 2:** Baseline characteristics of the overweight/obese study participants.

		Low-Dose Group (n = 20)		High-Dose Group (n = 32)		*p* Value ^a^
Variables		Mean	(SD)/(Range)	Mean	(SD)	
Age, y		44.0	(26–55)	43.7	(22–64)	0.91
Sex, N (%)	Male	4	(26.7)	5	(13.3)	0.72
	Female	16	(73.3)	27	(86.7)	
Height, cm		163.8	(8.0)	164.5	(9.4)	0.79
Weight, kg		85.45	(66–110)	90.9	(50–147)	0.42
BMI, kg/m^2^		31.8	(26–40)	33.3	(25–52)	0.65

^a^ Between-group differences (low dose vs. high dose) in the parameters were assessed by unpaired *t*-test (parametric) and Mann–Whitney U test (nonparametric) for continuous variables and Fisher’s Exact Test (2-sided) for categorical variables.

**Table 3:** Metabolic, hematological, and biochemical parameters before and after 10 days of NRE intervention in healthy participants.

	Low-Dose Group ^a^ (n = 15)				High-Dose Group ^a^ (n = 15)				Between Group *p* Value ^d^		
	Baseline	Day 10	Δ ^b^	*p* Value ^c^	Baseline	Day 10	Δ ^b^	*p* Value ^c^	Baseline	Day 10	Δ
**Variables**											
SBP, mmHg	111.9 (10.6)	109.9 (8.9)	−2.0 (4.6)	0.11	107.3 (10.3)	108.7 (9.9)	1.3 (6.4)	0.43	0.25	0.73	0.20
DBP, mmHg	63.7 (21.0)	63.5 (20.3)	−0.2 (23.9)	0.61	60.7 (10.3)	60 (11.3)	−0. 7 (2.6)	0.33	0.16	0.56	0.90
Pulse rate	74.7 (5.5)	73.5 (5.5)	−1.3 (4.2)	0.26	76.7 (9.1)	77.9 (9.1)	1.1 (6.9)	0.53	0.47	0.12	0.26
**Hematological Parameters**											
RBC, 10^12^/L	4.8 (0.5)	4.7 (0.7)	−0.1 (0.6)	0.62	4.3 (0.4)	4.5 (0.5)	0.2 (0.4)	0.05	0.002	0.37	0.10
WBC, 10^9^/L	6.6 (2.2)	6.3 (1.6)	−0.4 (1.0)	0.16	6.8 (1.7)	6.5 (1.7)	−0.3 (1.5)	0.45	0.80	0.67	0.87
Hb, gm/dL	13.4 (0.8)	13.5 (0.8)	0.1 (1.2)	0.75	12.2 (1.1)	12.7 (0.7)	0.5 (1. 1)	0.10	0.003	0.009	0.37
Ht, %	37.6 (7.4)	38.5 (2.3)	0.9 (7.2)	0.62	36.7 (2.9)	37.2 (2.8)	0.5 (2.6)	0.46	0.65	0.17	0.83
PLT, 10^9^/L	291.3 (73.4)	308 (52.1)	6.7 (102.5)	0.54	251.9 (55.8)	287.6 (41.8)	35.7 (64.7)	0.05	0.11	0.25	0.55
RBS, mmol/L	4.4 (0.5)	4.2 (0.5)	−0.1 (0.9)	0.55	4.9 (0.7)	4.7 (0.6)	−0.1 (0.9)	0.62	0.04	0.02	0.95
**Liver Function Test**											
ALT, UI/L	8.4 (3.3)	6.8 (2.3)	−1.6 (2.5)	0.03	8.7 (4.6)	8.5 (3.4)	−0.1 (5.3)	0.92	0.86	0.12	0.34
AST, UI/L	13.4 (2.8)	14.9 (3.6)	1.5 (3.5)	0.13	21.3 (6.3)	19.7 (4.2)	−1.6 (7.5)	0.42	<0.001	0.002	0.17
ALP, UI/L	38.7 (10.8)	45.2 (11.6)	6.5 (14.1)	0.09	53.4 (12.1)	52.1 (12.6)	−1.3 (7.5)	0.50	0.002	0.132	0.08
GGT, UI/L	15.5 (5.8)	16.7 (10.4)	0.5 (6.9)	0.21	11.6 (6.9)	11.9 (5.2)	0.3 (4.5)	0.78	0.10	0.05	0.93
BT, μmol/L	9.1 (7.7)	8.9 (7.1)	−0.1 (5.9)	0.65	8.1 (4.1)	6.3 (2.8)	−1.7 (4.5)	0.16	0.67	0.21	0.41
BD, μmol/L	1.9 (1.3)	1.9 (1.1)	−0.1 (1.0)	1.00	1.7 (1.1)	1.5 (0.7)	−0.3 (1.4)	0.55	0.81	0.32	0.68
sAlb, g/L	42.4 (3.2)	42.1 (3.2)	−0.2 (3.4)	0.78	41.8 (4.2)	42.7 (2.6)	0.9 (2.9)	0.24	0.68	0.56	0.32
**Renal Function Test**											
sCr, μmol/L	63.4 (11.6)	61.6 (12.1)	−1.8 (4.4)	0.14	59.5 (6.1)	58.3 (5.4)	−1.2 (5.3)	0.39	0.27	0.35	0.74
BUN, mmol/L	4.8 (1.9)	4.5 (1.3)	−0.3 (1.4)	0.41	3.8 (1.1)	3.7 (1.2)	−0.1 (1.1)	0.68	0.08	0.08	0.97
**Lipid Profile**											
TC, mmol/L	4.3 (0.6)	4.3 (0.7)	0.05 (0.5)	0.69	4.3 (0.9)	4.4 (0.8)	0.1 (0.4)	0.23	0.92	0.70	0.66
TG, mmol/L	0.9 (0.3)	0.8 (0.3)	−0.1 (0.2)	0.07	0.8 (0.3)	0.9 (0.3)	0.02 (0.2)	0.57	0.78	0.36	0.07
HDL, mmol/L	1.4 (0.6)	1.5 (0.5)	0.02 (0.5)	0.81	1.3 (0.2)	1.2 (0.2)	−0.05 (0.2)	0.40	0.31	0.09	0.57
LDL, mmol/L	2.4 (0.8)	2.4 (0.6)	0.01 (0.6)	0.92	2.7 (0.8)	2.8 (0.7)	0.2 (0.4)	0.18	0.43	0.13	0.46

^a^ Data are presented as mean (SD). ^b^ Mean difference before and after 10 days of NRE intervention (Day 10 − Baseline). ^c^ Within-group differences in the parameters before and after intervention were compared using paired *t*-test (parametric) and Wilcoxon Signed Rank test (nonparametric). ^d^ Between-group differences (low dose vs. high dose) in the parameters were assessed by using independent samples *t*-test (parametric and equal variances), Welch’s *t*-test (unequal variances), and Mann–Whitney U test (nonparametric).

**Table 4:** Metabolic, hematological, and biochemical parameters before and after 10 days of NRE intervention in overweight/obese participants.

	Low-Dose Group ^a^ (n = 20)				High-Dose Group ^a^ (n = 32)				Between Group *p* Value ^d^		
	Baseline	Day 10	Δ ^b^	*p* Value ^c^	Baseline	Day 10	Δ ^b^	*p* Value ^c^	Baseline	Day 10	Δ
**Variables**											
SBP, mmHg	115.2 (7.5)	111.5 (6.7)	−3.7 (6.7)	0.03	120.0 (13.7)	116.6 (10.9)	−3.4 (9.0)	0.04	0.13	0.03	0.82
DBP, mmHg	74.7 (6.4)	69.5 (10.9)	−5.2 (12.1)	0.08	68.1 (10.6)	63.1 (10.3)	−5.0 (9.5)	0.006	0.02	0.02	0.98
Pulse rate	76.5 (7.8)	76.6 (5.4)	0.05 (4.2)	0.96	78.8 (6.9)	78.2 (5.2)	−0.6 (3.8)	0.36	0.27	0.29	0.71
**Hematological Parameters**											
RBC, 10^12^/L	4.5 (0.8)	4.6 (0.8)	0.05 (0.4)	0.62	4.5 (0.5)	4.4 (0.5)	−0.04 (0.2)	0.52	0.80	0.48	0.78
WBC, 10^9^/L	7.3 (1.5)	7.4 (1.3)	0.05 (0.3)	0.42	6.6 (1.5)	7.0 (1.9)	0.4 (1.7)	0.22	0.12	0.47	0.60
Hb, gm/dL	11.3 (1.9)	11.3 (1.5)	−0.04 (0.6)	0.75	12.9 (1.7)	12.9 (1.6)	0.002 (0.7)	0.99	0.004	0.001	0.73
Ht, %	37.1 (5.4)	37.5 (5.9)	0.4 (3.9)	0.66	37.6 (5.2)	37.9 (4.9)	0.3 (1.9)	0.44	0.75	0.81	0.59
PLT, 10^9^/L	253.9 (52.2)	252.4 (53.3)	−1.4 (36.3)	0.65	245.0 (57.8)	230.5 (55.2)	−14.6 (38.9)	0.19	0.57	0.20	0.73
RBS, mmol/L	5.3 (1.8)	5.4 (1.9)	0.1 (0.5)	0.38	4.9 (0.8)	5.1 (0.7)	0.2 (0.8)	0.11	0.23	0.52	0.41
**Liver Function Test**											
ALT, UI/L	11.8 (5.5)	15.3 (15.0)	3.5 (13.2)	0.25	12.7 (10.8)	15.1 (8.1)	2.4 (8.4)	0.05	0.73	0.19	0.35
AST, UI/L	20.4 (14.3)	18.0 (7.6)	−2.4 (13.8)	0.44	21.8 (5.5)	22.5 (6.2)	0.7 (7.1)	0.57	0.008	0.02	0.73
ALP, UI/L	52.9 (17.3)	55.4 (16.9)	2.4 (12.1)	0.38	58.3 (19.2)	59.5 (24.3)	1.1 (20.8)	0.77	0.31	0.51	0.53
GGT, UI/L	22.1 (10.4)	24.3 (15.4)	2.2 (15.1)	0.52	24.2 (16.5)	23.3 (14.9)	−0.9 (5.7)	0.27	0.89	0.68	0.39
BT, μmol/L	10.6 (5.3)	9.5 (6.3)	−1.1 (6.7)	0.42	6.7 (2.8)	7.5 (4.2)	0.8 (2.7)	0.06	0.005	0.42	0.23
BD, μmol/L	1.8 (1.0)	1.9 (2.2)	0.07 (2.3)	0.51	1.4 (0.9)	1.5 (0.7)	0.03 (0.8)	0.62	0.03	0.72	0.23
sAlb, g/L	40.8 (2.5)	41.1 (2.6)	0.2 (2.5)	0.65	40.5 (2.4)	39.8 (7.3)	−0.8 (7.8)	0.58	0.68	0.44	0.57
**Renal Function Test**											
sCr, μmol/L	54.4 (15.5)	53.3 (16.6)	−1.1 (5.7)	0.38	60.2 (13.9)	59.0 (12.5)	−1.2 (5.3)	0.19	0.17	0.17	0.95
BUN, mmol/L	4.1 (0.9)	4.5 (1.5)	0.4 (1.3)	0.21	4.2 (1.3)	4.4 (1.1)	0.2 (0.9)	0.22	0.86	0.73	0.54
**Lipid Profile**											
TC, mmol/L	4.7 (0.9)	4.8 (1.1)	0.1 (0.6)	0.37	5.1 (0.9)	5.1 (0.9)	0.03 (0.5)	0.69	0.13	0.28	0.56
TG, mmol/L	1.4 (0.6)	1.4 (0.7)	−0.07 (0.3)	0.38	1.3 (0.5)	1.2 (0.5)	−0.1 (0.3)	0.004	0.58	0.32	0.56
HDL, mmol/L	1.1 (0.2)	1.1 (0.3)	−0.01 (0.2)	0.69	1.2 (0.3)	1.4 (0.3)	0.1 (0.1)	<0.001	0.15	0.004	0.003
LDL, mmol/L	2.9 (0.7)	2.9 (0.8)	0.08 (0.5)	0.52	3.2 (0.7)	3.2 (0.7)	−0.07 (0.5)	0.37	0.09	0.39	0.28

^a^ Data are presented as mean (SD). ^b^ Mean difference before and after 10 days of NRE intervention (Day 10 − Baseline). ^c^ Within-group differences in the parameters before and after intervention were compared using paired *t*-test (parametric) and Wilcoxon Signed Rank test (nonparametric). ^d^ Between-group differences (low dose vs. high dose) in the parameters were assessed by using independent samples *t*-test (parametric and equal variances), Welch’s *t*-test (unequal variances), and Mann–Whitney U test (nonparametric).

## Data Availability

**All** data generated from the study are available within this paper and in Supplementary Materials. Additional data that do not fall under ethical restrictions due to human subject involvement can be made available upon request to the corresponding author (S.S.).

## Supplementary Materials

The following supporting information can be downloaded at: https://www.mdpi.com/article/10.3390/nu15163649/s1, Table S1: Lipid profile in high-dose group of overweight/obese participants with BMI < 29.9 (n = 10); Table S2: Lipid profile in high-dose group of overweight/obese participants with BMI > 30.0 (n = 22); Table S3: Lipid profile in high-dose group of overweight/obese participants with age < 40 yrs (n = 13); Table S4: Lipid profile in high-dose group of overweight/obese participants with age > 40 yrs (n = 19); Table S5: Lipid profile in high-dose group of overweight/obese participants with age < 40 yrs, BMI < 29.9 (n = 7); Table S6: Lipid profile in high-dose group of overweight/obese participants with age < 40 yrs, BMI > 30.0 (n = 6); Table S7: Lipid profile in high-dose group of overweight/obese participants with age > 40 yrs, BMI < 29.9 (n = 6) and Table S8: Lipid profile in high-dose group of overweight/obese participants with age > 40 yrs, BMI > 30.0 (n = 13).
