# Supplementary material for: Exploring the Effects of Short-Term Daily Intake of Nitraria retusa Tea on Lipid Profile: A Pre-Post, Uncontrolled Pilot Study in Both Healthy and Overweight/Obese Adults
Source: Nutrients. 2023 Aug 20;15(16):3649. doi: 10.3390/nu15163649 (PMC10459123; doi:10.3390/nu15163649)
Supplement: Supplementary file 1 [file nutrients-15-03649-s001.zip › nutrients-2545546-supplementary.pdf]

Supplementary Data

Supplementary Table S1: Lipid profile in high-dose group of overweight/obese participants with BMI $\leq$ 29.9 (n = 10)

	Before	After	*p* value[a]
**TC, mmol/L**	4.78	4.92	0.19
**TG, mmol/L**	1.23	1.20	0.41
**HDL, mmol/L**	1.21	1.30	0.051
**LDL, mmol/L**	3.01	3.07	0.28

[a]Within-group differences in the parameters before and after intervention were compared using paired t-test (parametric)

Supplementary Table S2: Lipid profile in high-dose group of overweight/obese participants with BMI $\geq$ 30.0 (n = 22)

	Before	After	*p* value[a]
**TC, mmol/L**	5.21	5.19	0.44
**TG, mmol/L**	1.41	1.23	0.006
**HDL, mmol/L**	1.28	1.43	< 0.001
**LDL, mmol/L**	3.33	3.19	0.11

[a]Within-group differences in the parameters before and after intervention were compared using paired t-test (parametric)

Supplementary Table S3: Lipid profile in high-dose group of overweight/obese participants with age < 40 yrs (n = 13)

	Before	After	*p* value[a]
**TC, mmol/L**	4.72	4.81	0.23
**TG, mmol/L**	1.24	1.03	0.011
**HDL, mmol/L**	1.14	1.26	0.005
**LDL, mmol/L**	3.08	3.07	0.49

[a]Within-group differences in the parameters before and after intervention were compared using paired t-test (parametric)

Supplementary Table S4: Lipid profile in high-dose group of overweight/obese participants with age > 40 yrs (n = 19)

	Before	After	*p* value[a]
**TC, mmol/L**	5.32	5.32	0.48
**TG, mmol/L**	1.43	1.36	0.22
**HDL, mmol/L**	1.34	1.47	0.001
**LDL, mmol/L**	3.33	3.21	0.102

[a]Within-group differences in the parameters before and after intervention were compared using paired t-test (parametric)

Supplementary Table S5: Lipid profile in high-dose group of overweight/obese participants with age < 40 yrs, BMI $\leq$ 29.9 (n = 7)

	**Before**	**After**	***p* value[a]**
**TC, mmol/L**	4.67	4.78	0.22
**TG, mmol/L**	1.04	0.92	0.025
**HDL, mmol/L**	1.24	1.32	0.11
**LDL, mmol/L**	2.94	3.03	0.25

[a]Within-group differences in the parameters before and after intervention were compared using paired t-test (parametric)

Supplementary Table S6: Lipid profile in high-dose group of overweight/obese participants with age < 40 yrs, BMI $\geq$ 30.0 (n = 6)

	**Before**	**After**	***p* value[a]**
**TC, mmol/L**	4.79	4.84	0.39
**TG, mmol/L**	1.47	1.16	0.054
**HDL, mmol/L**	1.03	1.196	0.006
**LDL, mmol/L**	3.23	3.12	0.36

[a]Within-group differences in the parameters before and after intervention were compared using paired t-test (parametric)

Supplementary Table S7: Lipid profile in high-dose group of overweight/obese participants with age > 40 yrs, BMI $\leq$ 29.9 (n = 6)

	**Before**	**After**	***p* value[a]**
**TC, mmol/L**	5.37	5.53	0.27
**TG, mmol/L**	1.51	1.57	0.41
**HDL, mmol/L**	1.22	1.30	0.08
**LDL, mmol/L**	3.47	3.46	0.49

[a]Within-group differences in the parameters before and after intervention were compared using paired t-test (parametric)

Supplementary Table S8: Lipid profile in high-dose group of overweight/obese participants with age > 40 yrs, BMI $\geq$ 30.0 (n = 13)

	**Before**	**After**	***p* value[a]**
**TC, mmol/L**	5.30	5.22	0.26
**TG, mmol/L**	1.39	1.26	0.028
**HDL, mmol/L**	1.39	1.55	0.005
**LDL, mmol/L**	3.28	3.09	0.082

[a]Within-group differences in the parameters before and after intervention were compared using paired t-test (parametric)
